# Phenotype-Driven Virtual Panel Is an Effective Method to Analyze WES Data of Neurological Disease

**DOI:** 10.3389/fphar.2018.01529

**Published:** 2019-01-09

**Authors:** Xu Wang, Xiang Shen, Fang Fang, Chang-Hong Ding, Hao Zhang, Zhen-Hua Cao, Dong-Yan An

**Affiliations:** ^1^Department of Neurology, Beijing Children’s Hospital, National Centre for Children’s Health, Capital Medical University, Beijing, China; ^2^Running Gene Inc., Beijing, China

**Keywords:** WES, phenotype-driven, virtual panel, rare disease, annotation

## Abstract

**Objective:** Whole Exome Sequencing (WES) is an effective diagnostic method for complicated and multi-system involved rare diseases. However, annotation and analysis of the WES result, especially for single case analysis still remain a challenge. Here, we introduce a method called phenotype-driven designing “virtual panel” to simplify the procedure and assess the diagnostic rate of this method.

**Methods:** WES was performed in samples of 30 patients, core phenotypes of probands were then extracted and inputted into an in-house software, “Mingjian” to calculate and generate associated gene list of a virtual panel. Mingjian is a self-updating genetic disease computer supportive diagnostic system that based on the databases of HPO, OMIM, HGMD. The virtual panel that generated by Mingjian system was then used to filter and annotate candidate mutations. Sanger sequencing and co-segregation analysis among the family were then used to confirm the filtered mutants.

**Result:** We first used phenotype-driven designing “virtual panel” to analyze the WES data of a patient whose core phenotypes are ataxia, seizures, esotropia, puberty and gonadal disorders, and global developmental delay. Two mutations, c.430T > C and c.640G > C in PMM2 were identified by this method. This result was also confirmed by Sanger sequencing among the family. The same analysing method was then used in the annotation of WES data of other 29 neurological rare disease patients. The diagnostic rate was 65.52%, which is significantly higher than the diagnostic rate before.

**Conclusion:** Phenotype-driven designing virtual panel could achieve low-cost individualized analysis. This method may decrease the time-cost of annotation, increase the diagnostic efficiency and the diagnostic rate.

## Introduction

Rare Disease is defined as disease affected less than one in 2000 citizens in Europe, or less than one in 1250 in the United States (Schieppati et al., 2008). Rare diseases often start in childhood and accompanied by multisystem disorders which affect life quality of patients (Dodge et al., 2011; Elliott and Zurynski, 2015; Wright et al., 2018). Moreover, 33% of rare disease children die before 5 years old (Wright et al., 2018). There are now approximately 10,000 rare diseases (Elliott and Zurynski, 2015), about 4 of 5 rare disease patients are thought to have a genetic base (Plaiasu et al., 2010; Dodge et al., 2011) especially monogenic disorder (Stolk et al., 2006). For some rare disorders such as tuberous sclerosis complex, phenotypes may vary among individuals due to heterogeneous manifestations. Merely diagnosis based on clinical presentations could be a great challenge (Bai et al., 2017). Hence, gene sequencing for the pathogenic genes is vital for understanding the cause of diseases.

The mainstream of gene sequencing includes genomic microarrays, Sanger sequencing and Next-Generation sequencing (NGS). Genomic microarrays are low-resolution method for detection of 50∼100 kb copy number variation (Speicher and Carter, 2005). For small insertion or deletion less than 50 kb, Sanger sequencing and NGS could fulfill the task. Sanger sequencing, due to limited throughput, is only used when a specific gene is selected. Different diseases could have similar clinical presentations such as ataxia and mental retardation. At the same time, a disease may be caused by various genes. It is difficult to determine the pathogenic gene in every patient to perform Sanger sequencing. NGS offers much higher throughput that can facilitate sequencing up to 1000s of gene once. In addition, since sheared DNA is sequenced parallelly multiple times, therefore lower error rate is achieved compared to Sanger sequencing. Moreover, the recent study showed that NGS could also be used to detect Copy Number variation that larger than 100 kb (de Ligt et al., 2013; Feng et al., 2017). Therefore, it has been increasingly used in rare disease diagnosis.

For NGS, the range of detection object could vary from multiple disease-associated genes (gene panel), whole exome (Whole-Exome Sequencing) to whole genome (Whole-Genome Sequencing). For gene panel, various genes affected several similar diseases or diseases in the same system could be detected at the same time. Since it focuses on the specific genes, the data size is generally smaller than Whole-Exome Sequencing (WES) and Whole-Genome Sequencing (WGS), the result is easy to analyze and interpret. Although convenient, the gene list of a particular panel is constant; meanwhile, the discovery of disease-associated gene is developing. The newly discovered gene on one hand cannot be added to the already made panel, and further analysis cannot be performed. On the other hand, updating gene list every day is, however, impractical, costly and with less sense. Gene panels is at present insufficient for detection and is not recommended by most of the genetics and clinicians (Biesecker and Green, 2014; Wenger et al., 2017; Ewans et al., 2018; Jin et al., 2018).

WGS, mostly based on Illumina technology, is the sequencing method covers most part of the human genome. Although easy to perform, it is costly and time consuming to analyze and interpret data. On average, 3–4 million mutations could be discovered in each individual (Ashley et al., 2010; Lupski et al., 2010; Roach et al., 2010; Sobreira et al., 2010; Bainbridge et al., 2011). In the meantime, the mutations in the intronic region except for the ones near splicing sites are hard to predict the relative risk of phenotype, since the function of the intronic gene is still mostly undiscovered, and the mutation frequency in the intron is considerably high (Tabor et al., 2002; Abecasis et al., 2010). It is hard to estimate which mutation is deleterious. Research also presented that WGS has limited significance at the present stage (Alfares et al., 2018). By contrast, the exome represents 1–2% protein-coding gene of the whole genome thus more exomes could be sequenced per run (Gilissen et al., 2012). The result of WES is more accessible to interpret since non-synonymous mutations in the coding region could directly lead to amino acid change then affect the protein structure and function. This method could also help identify not only the unknown pathological mutations but also the undiscovered mutations (Liu et al., 2012). Re-analysis of WES data was also proved to significantly increase the diagnostic rate (Alfares et al., 2018). The cost of WES is also much lower than WGS (Gilissen et al., 2012) at present. Although the number of variants is cut down to the range between 20,000 and 50,000 (Ashley et al., 2010; Lupski et al., 2010; Roach et al., 2010; Sobreira et al., 2010; Bainbridge et al., 2011; Gilissen et al., 2012), it is still difficult to analyze and identify the pathogenicity of every variant, especially for detection of single case because of lower efficiency and time consuming. Meanwhile, due to the analysis strategy with less-efficacy, the diagnostic rate of WES with unspecific analysis was relatively low, approximately 25–30% (Yang et al., 2013; Lee et al., 2014; Shashi et al., 2016).

After carrying out, investigating and studying WES in clinic for many years, the combination of clinical information and gene sequencing is increasingly suggested in disease diagnosis (Jin et al., 2018). Here, we developed a method called “Phenotype-driven designing virtual panel,” a method that concentrates in analysing the genes of diseases with related phenotypes. The gene lists of phenotype-associated diseases were generated by a system called “Mingjian.” After inputting all phenotypes of the patient, the system will automatically list the associated genes and rank the gene by the corresponding number of phenotypes. This method is proved to improve the diagnostic rate significantly in our further test.

## Methods

### Whole-Exome Sequencing

Proband DNA was sequenced to discover the causal gene. DNA was isolated from peripheral blood using a DNA Isolation Kit (Bioteke, AU1802). 1ug genomic DNA was fragmented into 200–300 bp length by Covaris Acoustic System. The DNA fragments were then processed by end-repairing, A-tailing and adaptor ligation, a 4-cycle pre-capture PCR amplification, targeted sequences capture. Captured DNA fragments were eluted and amplified by 15 cycle post-capture PCR. The final products were sequenced with 150 bp paired-end reads on Illumina HiSeq X platform according to the standard manual.

**FIGURE 1:**
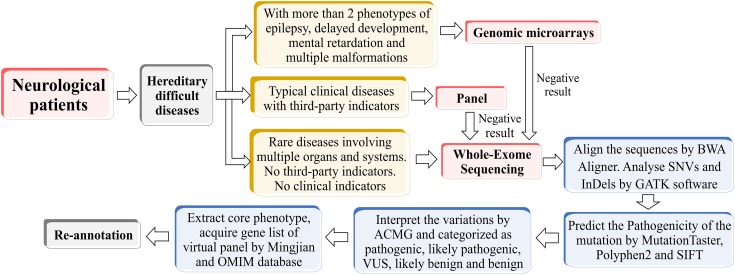
The flow chart of phenotype-driven designing virtual panel.

The raw data converted by HiSeq X were filtered and aligned against the human reference genome (hg19) using the BWA Aligner^1^. The single-nucleotide polymorphisms (SNPs) were called by using the GATK software (Genome Analysis ToolKit) ^2^. Variants were annotated using ANNOVAR ^3^. Effects of single-nucleotide variants (SNVs) were predicted by SIFT, Polyphen-2, and MutationTaster programs. All variants were interpreted according to the standards for interpretation of sequence variations recommended by ACMG and categorized to be pathogenic, likely pathogenic, variants of unknown clinical significance (VUS), likely benign and benign. The associated phenotypic features of candidate genes were analyzed against the patient’s phenotype. Core phenotypes were extracted and used to acquire a gene list of the virtual panel by OMIM database^4^ and Mingjian (211.149.234.157/login). Re-annotation was conducted according to the virtual panel. The whole process was shown in Figure 1.

### Sanger Sequencing

The candidate causal genes discovered via WES were then confirmed by Sanger sequencing, co-segregation analyses among the family were also conducted. The primers were designed using Primer Premier 5.0 (Premier Biosoft), PCR was carried out to amplify the fragments covering the mutated sites. The PCR products were further purified with Zymoclean PCR Purification Kit and then sequenced by ABI 3730 DNA Sequencer. Sanger sequencing results were analyzed by Chromas Lite v2.01 (Technelysium Pty Ltd., Tewantin, QLD, Australia).

## A Case of a Diagnostic Odyssey

**FIGURE 2:**
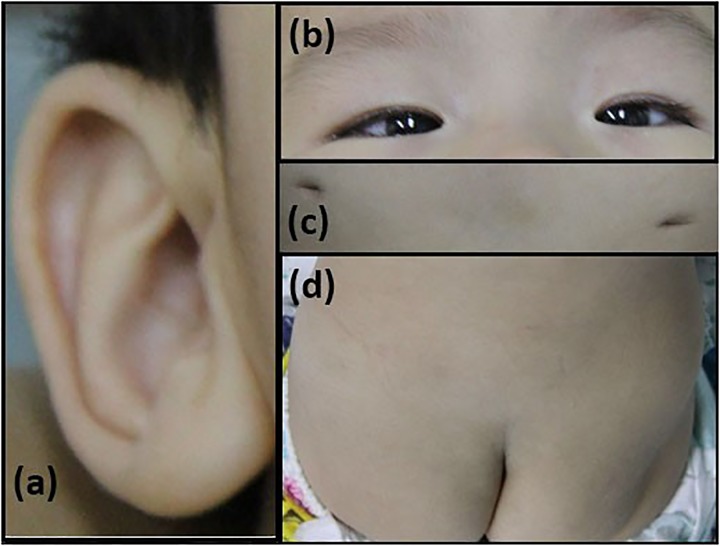
Phenotype of the patients. **(a)** large ear; **(b)** internal strabismus; **(c)** inverted nipples; **(d)** fat pad in the buttock.

**FIGURE 3:**
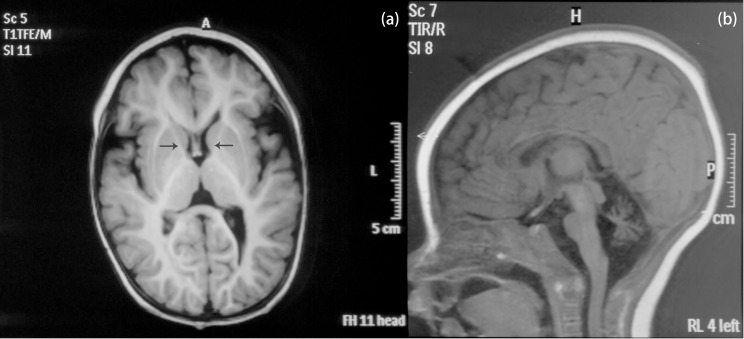
MRI result of the patient. **(a)** Coronal T1W image. Black arrows pointed the anterior limb of the internal capsule. The faint hyperintense signal of T1W indicated delayed myelination of the patient. **(b)** Sagittal T1W image. The patient presented cerebellar atrophy.

**FIGURE 4:**
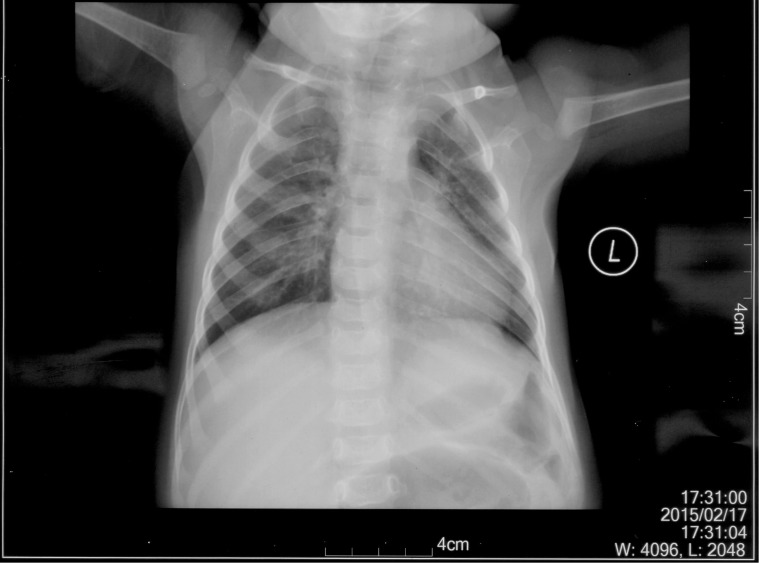
Chest CT result of the patient. It is shown that the patient had spine kyphosis.

**FIGURE 5:**
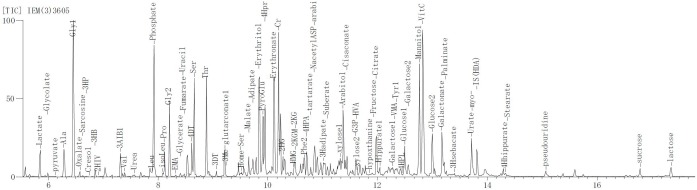
The mass spectrum result of the metabolite in urine. The result of the urine metabolite indicated liver abnormality of patient.

**FIGURE 6:**
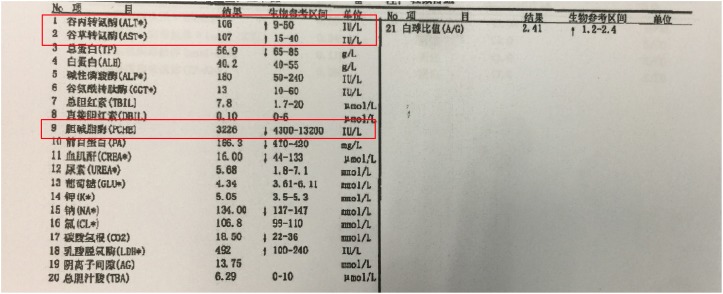
The blood test result of the patient’s serum. Alleviation of alanine aminotransferase (ALT) and Aspartate aminotransferase (AST) and deduction of plasma cholinesterase indicated liver dysfunction of the patient.

The patient is an 8 months old boy who was born to a normal non-consanguineous Han family by normal vaginal delivery at full-term. He had tonic seizure epilepsy with sustaining state when he first came to our hospital. His symptoms get alleviated obviously after taking levetiracetam 40 mg/kg per day. The milestone development and comprehensive development of the patient was also delayed. Physical examination: the head circumference of the patient was 41 cm, anterior fontanel was 1^∗^1 cm. He had internal strabismus but could chase light, he also presented large ear, low nose, inverted nipples, low muscle tension with muscle strength-4, weak tendon reflex, poor head control, round back, fat pad in buttock, bilateral cryptorchidism and short penis. His body always leaned forward when sitting (Figure 2). He could not open his mouth or speak actively. He could neither grab things initiatively. Laboratory result: MRI result presented cerebellar atrophy and delayed myelination (Figure 3); chest CT showed spine kyphosis (Figure 4); EMG result showed neurogenic damage; the LC-MS/MS result of blood (Table 1), GC-MS result of urine (Figure 5) and blood test of patient’s serum (Table 2 and Figure 6) indicated abnormal liver function.

**Table 1:** Mass spectrum result of patient’s blood.

	Result of patient		Reference for children (6 months to 1 year old)	Ratio
His	99.916	↑	0.00–79.30	1.260
Tyr	19.083	↓	19.40–79.40	0.240
Thr	87.512	↑	22.00–64.20	1.363
Phe/Tyr	1.980	↑	0.23–1.20	1.650
C5DC	0.099	↑	0.00–0.08	1.232
C0/C2	0.568	↓	0.82–2.40	0.237


*Enhancement of His, Thr, Phe/Tyr, C5DC and regression of Tyr and C0/C2 indicated liver dysfunction of the patient.*

**Table 2:** Blood test result of the patient’s serum.

	Result		Reference	Unit
ALT	106	↑	9–50	IU/L
AST	107	↑	15–40	IU/L
PCHE	3226	↓	4300–13200	IU/L


*Alleviation of alanine aminotransferase (ALT) and Aspartate aminotransferase (AST) and deduction of plasma cholinesterase indicated liver dysfunction of the patient.*

**FIGURE 7:**
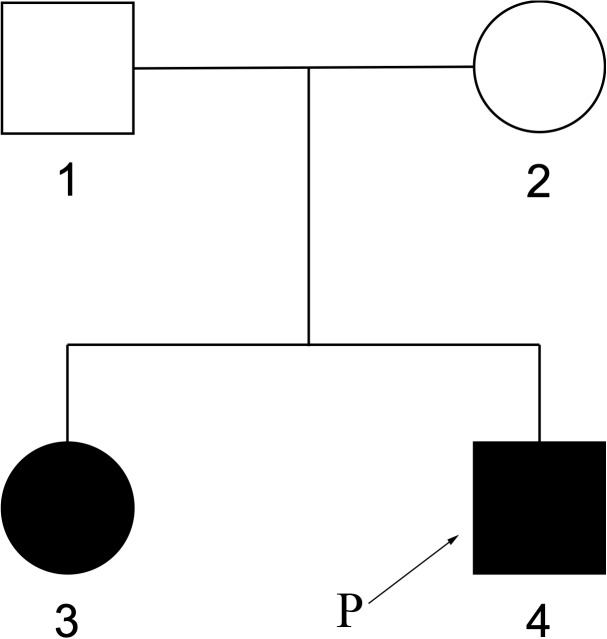
Pedigree of family members. The elder sister of the patient also had similar phenotypes.

The elder sister of the patient, 8 years old, also shows somehow similar phenotypes. At 2 years of age, she started to have tonic epilepsy and ataxia, mental retardation, so far can only speak 2–3 words phrase. The pedigree was shown in Figure 7.

The clinical presentation involved multiple systems and thus, even he has got treated at many hospitals and screened by existing detection methods, the disease was still unclear.

## Results

### The Gene List of Phenotype-Driven Virtual Panel

Extracting and inputting the core phenotypes: Ataxia, Seizures, Esotropia, Puberty and Gonadal disorders, Global developmental delay, Autosomal recessive (inheritance pattern). The gene list exported by Mingjian is listed in Table 3.

**Table 3:** Gene list exported by Software Mingjian according to the inputting core phenotypes.

Number of consilient phenotypes	Gene lists
6	PMM2 CEP290
5	GBA, POLG, GP1BB, HSD17B4, PEX1, PEX6, ERCC2 BCS1L, DOCK8, PEX10, TCF4, PEX12, ERCC6, RRM2B, PEX26, PEX2, ERCC4, PEX16, GRIN2B, PEX5, ERCC1, WDR73, PEX3, K1F1A, PEX14, PEX19, PEX11B, ADGRG1, C100RF2
4	ABCD1, SCN1A, ABCC8, PTS, SURF1, BTD, NPC1,GCH1, ASL, CDKL5, ASS1, ATM, PRF1, GAMT, PDHA1, CPS1, OFD1, PLA2G6, SOX10, ETHE1, GJA1, ADSL, PROKR2, FGFR1, PPT1, FKRP, OTX2, POMGNT1, NPC2, SCO2, SIL1, BBS2, UNC13D, POMT1, TBX1, BBS1, STXBP1, BBS10, NDUFS4, ALMS1, GJC2, STXBP2, NPHP1, BRAF,HESX1, NDUFV1, ECHS1, MKKS, ERCC8, GMPPB, BBS12, NDUFS8, TUBB2B, POLR1C, COQ2, MKS1, SUCLG1, FMR1, BBS4, POLR3B, SPR, RAB3GAP1, ADLH5A1, RAF1, NDUFAF2, SDHA, EDNRB, CC2D2A, RARS2, ARL6, TSEN54, SUOX, SLC17A5, MBD5, POMT2, SCN2A, MMADHC, SCN9A, MFSD8, NDUFS2, SLC25A1, BBS7, POLR3A, PCNT, NDUFS6, EDN3, PDHX, PNKP, BBS9, WWOX, PSAP, DPM1, DYRK1A, NDUFA1, PET100, TTC8, ALG6, FKTN, DLD, NDUFS1, TMEM216, BBS5, SDCCAG8, SLC19A3, SYNGAP1, HIBCH, NDUFS7, COX6B1, NDUFAF1, MTFMT, SLC6A19, ALG1, LARGE, ERCC3, NOTCH1, CTC1, KCNJ10, GLI2, IFT172, TRIM32, NDUFS3, LIPT1, DOCK6, DYNC1H1, NDUFAF3, SCO1, NDUFB9, SLC46A1, NDUFA2, TMEM138, TMEM138, NDUFB3, DLL4, NDUFAF5, TTC19, GABRA1, COA3, FOXRED1, STX11, COX10, SLC25A4, DEAF1, ACO2, NDUFV2, B3GALNT2, GRIN1, APOPT1, NUBPL, TSFM, CDH15, NDUFA12, CYC1, WDPCP, RAB3GAP2, RFT1, TACO1, COX14, TMEM231, TMEM237, NDUFA11, GRM1, NDUFAF6, ZNF423, RPIA, KIRREL3, ATP5A1, NDUFA4, IFT27, COMT, PDSS2, NDUFAF4, UQCC2, LZTFL1, EOGT, UQCRQ, NDUFA9, COX15, NDUFA10, UQCRC2, UQCC3, DHFR, BBIP1, PDP1, CACNG2, PLXND1, COX20, ARHGAP31, RBPJ, EPB41L1, NIN, CTDP1, MYO5A, UQCRB, NAT8L, LYRM7, FASTKD2, ZNF592, C5ORF42, ND3, ND2, TRNV, ND5, ND4, ND1, ATP6, CYTB, ND6, TRNL1, COX2, COX3, TRNK, RNU4ATAC, COX1, TRNW
3	OTC, DMD, PROC, SDHB… altogether 441 genes
2	GLA, PAH, GCK, GALT… altogether 543 genes
1	HBB, LDLR, MLH1… altogether 1427 genes


### Result of Whole-Exome Sequencing

**FIGURE 8:**
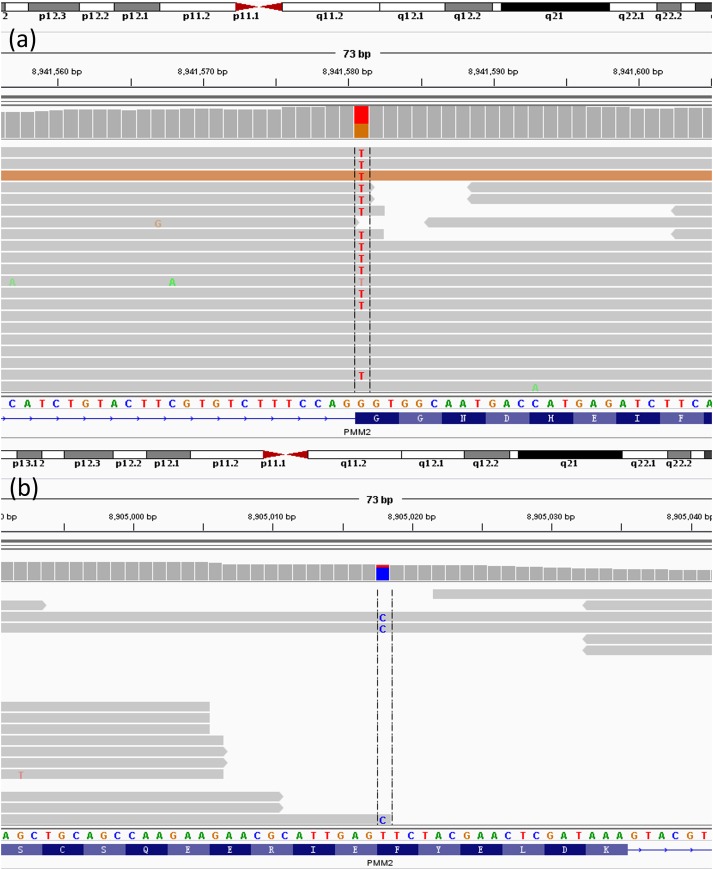
Next-Generation result of patient. The result shows the two heterozygous mutations in the PMM2 gene. **(a)** c.640G > C; **(b)** c.430T > G.

Analysing the gene from gene list generated by Mingjian according to the core phenotypes, two heterozygous mutations in PMM2 gene had been found, c.430T > C in exon 5 (chr16:8905018 T > C) and c.640G > C in exon 8 (chr16:8941581G > C). These nucleotide substitutions would result in alterations in amino acid, F144L and G214R, respectively (Figure 8).

**FIGURE 9:**
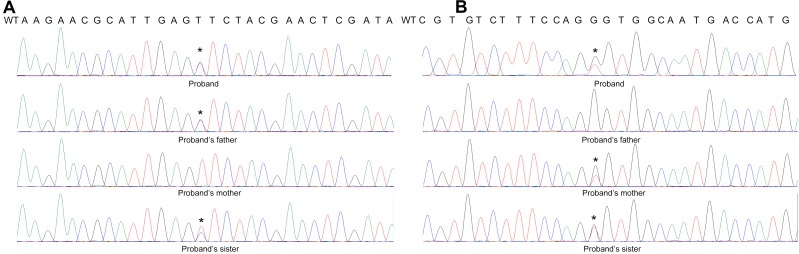
Sanger Sequence result of the patient’s family. The result shows that **(A)** the proband’s father was the heterozygous carrier of the c.430T > C mutation, while **(B)** the proband’s mother carried the c.640G > C mutation. The proband’s sister is also the carrier of the compound heterozygous mutations.

Further Sanger Sequencing result showed the proband’s father is the heterozygous carrier of the c.430T > C mutation, while the proband’s mother carries the c.640G > C mutation. The proband’s sister with the same clinical presentation also carries all these two mutations. Thus, the proband is the compound heterozygous for the PMM2 p.F144L/p.G214R mutations (Figure 9).

**FIGURE 10:**
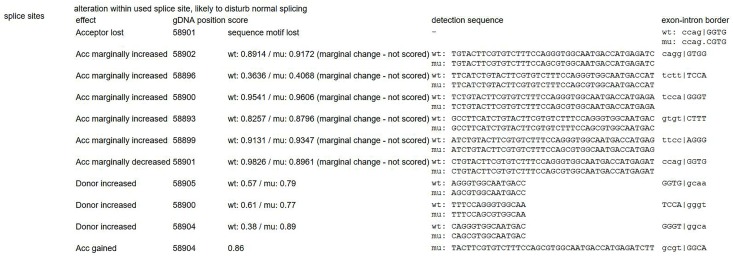
Indicated splicing change by MutationTaster (Schwarz et al., 2014). This mutation might disturb the exon-intron border.

Mutation p.F144L is a pathologic mutation that has been reported before. This mutation could create a new site for restriction enzyme SacI causing extra splicing (Kondo et al., 1999). Another mutation p.G214R has not been reported before, however, there is another reported disease-causing mutation at the same position (c. 640G > A, G214S) (Schollen et al., 2002; Vicario et al., 2017). Since this mutation is absent from controls (PM2), detected in trans with a pathogenic variant (PM3), located at the same position with a reported pathogenic missense change (PM5), this variant was classified as “likely pathogenic” according to ACMG guidelines (Richards et al., 2015). Prediction of this mutation by MutationTaster, Provean and SIFT also turned out to be disease causing (probability > 0.99), deleterious (score = -7.66) and damaging (score = 0), respectively. The result of MutationTaster (Schwarz et al., 2014) also indicated splice site change caused by the mutation (Figure 10), however mRNA experiment was not successfully performed to prove it.

### Result of Other Patients

To assess the diagnostic rate of this method, “phenotype-driven virtual panel,” we decided to use the same method to analyze more neurological patients.

#### Clinical Information of the Patients

The clinical phenotypes of 29 patients were listed in Table 4.

**Table 4:** Phenotype of 29 patients with neurological diseases.

Patient No.	Age range	Phenotypes
1	2–4	Gait instability, worse in the dark; dystonia
2	0–2	Intellectual disability; hearing abnormity; congenital cataract; talipes equinovarus; brain atrophy
3	>4	Intellectual disability; dark skin; abnormal facial shape
4	2–4	Global development delay; autism; optic atrophy; gait disturbance
5	0–2	Intellectual disability; abnormal facial shape; dystonia; muscle weakness
6	>4	Seizures; epileptic encephalopathy; intellectual disability; microcephaly
7	2–4	Intellectual disability; abnormal facial shape; autism
8	>4	Intellectual disability; short stature
9	0–2	Seizures; Intellectual disability; microcephaly; abnormal ear morphology
10	2–4	Seizures; Intellectual disability; abnormality of metabolism/homeostasis
11	0–2	Intellectual disability; seizures
12	0–2	Intellectual disability; hypertonia; esotropia; abnormality of metabolism/homeostasis
13	>4	Delayed gross motor development; Intellectual disability; agenesis of corpus callosum
14	>4	Intellectual disability; cryptorchidism; Short stature
15	2–4	Intellectual disability; autism
16	2–4	Intellectual disability; autism
17	0–2	Intellectual disability; elevated urine guanidinoacetic acid
18	2–4	Delayed gross motor development; Intellectual disability, ulnar claw
19	0–2	Seizures; global development delay; high palate
20	2–4	Seizures
21	>4	Intellectual disability; autism
22	>4	Seizures; glutaric aciduria
23	0–2	Seizures (VB6 improvement); intellectual disability; dyspepsia
24	0–2	Intellectual disability; microcephaly; abnormality of metabolism/homeostasis
25	0–2	Seizures; intellectual disability; vitamin B6 deficiency
26	2–4	Intellectual disability; cerebellar atrophy
27	2–4	Seizure; arachnoid cyst
28	0–2	Intellectual disability; autism
29	0–2	Multiple-malformation; dystonia


Patients were collected from the neurology department of Beijing Children’s Hospital. Of the 29 patients, 19 patients (65%) are male, 10 patients (35%) are female. The ages range from 4 months to 17 years 6 months. Most patients have an intellectual disability. More precise clinical information, phenotypes and gene sequencing result were available in Supplementary Material.

#### Sequencing Results of Patients

The gene sequencing results of these 29 patients was listed in Table 5.

**Table 5:** Gene sequencing result of 29 patients with neurological diseases.

Patient No.	Gene	Position	Nucleotide variant	Protein variant	Inheritance pattern
1	COMP	chr19:18896846	c.1418A > G	p.D473G	AD
2	DYNC1H1 CRYBB2	chr14:102499762 chr22:25627591	c.10354G > A c.470C > G	p.A3452T p.P157R	AD
3	ADNP	chr20:49520469	c.64dupA	p.I22fs	AD
4	SYNE2	chr14:64675492	c.18218T > C	p.I6073T	AD
5	COL6A3	chr2:238245098 chr2:238275918	c.8645C > T c.4912G > A	p. P2882L p.A1638T	AD/AR
6	CHD2	chr15:93498742	c.1809G > T	p.K603N	AD
		chr15:93498743	c.1809+1G > T	Splicing	
7	HCFC1	chrX:153220254	c.3596G > C	p.R1199P	XR
8	SUCLG1	chr2:84652709 chr2:84660557	c.884G > A c.592A > G	p.V282I p.I198V	AR
9	SMARCAL1	chr2:217285085	c.926G > A	p.S309N	AR
		chr2:217332750	c.2225C > T	p.T742M	
10	MTHFR	chr1:11856378	c.665C > T	p.A222V	AR
		chr1:11863038	c.136C > T	p.R46W	
11	CDKL5	chrX:18582616	c.119C > T	p.A40V	XD
12	PDGFRB	chr5:149512504	c.936G > C	p.E312D	AD
13	TUBA1A	chr12:49579133	c.1016G > T	p.R339L	AD
14	SMC1A	chrX:53441721	c.331T > G	p.F111V	XD
15	HUWE1	chrX:53578276	c.9047A > C	p.E3016A	Unknown
	MAPT	chr17:44060834	c.664C > G	p.R222G	AD
16	MECP2	chrX:153296153	c.1162C > T	p.P388S	XD/XR
	KCNC1	chr11:17793707	c.1066G > A	p.V356M	AD
17	DYNC1H1 XDH	chr14:102463472 chr2:31596756 chr2:31598377	c.3665A > G c.1669G > A c.1471G > A	p.N1222S p.D557N p.A491T	AD AR
18	IGHMBP2	chr11:68702842 chr11:68704545	c.1708C > T c.2598_2599del	p.R570X p.K866Sfs	AR
19	CHD2	chr15:93563380	c.5045A > G	p.D1682G	AD
	CSF1R	chr5:149433641	c.2909_2910insATCA	p.Q970fs	AD
	EZH2	chr7:148544336	c.55G > A	p.V19I	AD
20	ND	ND	ND	ND	ND
21	ND	ND	ND	ND	ND
22	ND	ND	ND	ND	ND
23	ALDH7A1	chr5:125919644	c.454A > G	p.I152V	AR
24	ND	ND	ND	ND	ND
25	ND	ND	ND	ND	ND
26	SLC22A5	chr5:131728257	c.1400C > G	p.S467C	AR
27	ND	ND	ND	ND	ND
28	ND	ND	ND	ND	ND
29	ND	ND	ND	ND	ND


*Twenty one of Twenty nine patients have been sequenced with a suspected gene, however, 2 of them have not corresponded with the inheritance pattern, i.e., autosome recessive gene with only one mutation. AD, autosomal dominant; AR, autosomal recessive; XD, X-linked dominant; XR, X-linked recessive; ND, not detected with related mutations.*

## Discussion

Rare diseases, especially the ones involving multisystem are challenges for clinical diagnosis. For example, the PMM2 case described here involves not only the nervous system but also muscle, gonad, liver, spine, etc. It is hard to distinguish the fundamental factors of the pathogenesis by only examine clinical symptoms. Judging merely based on the clinical information, misdiagnosis was definitely not a rare event, especially in the generation without gene detection. A patient in our hospital who was previously diagnosed as Crouzon syndrome was finally proved to be Cytochrome P450 oxidoreductase deficiency by NGS (Hao et al., 2018). Misdiagnosis can result in a completely different treatment and might have possibility in leading deterioration. The efficacy of treatment might also be affected when the optimal treatment time is missed. Thus, gene sequencing is essential in the diagnosis of rare diseases.

Core phenotypes of patients with the neurological inherited disease are similar, i.e., ataxia, seizures, esotropia, global developmental delay, puberty and gonadal disorders in this case. It is almost impossible to only rely on clinicians’ experience to diagnose and determine candidate genes. Evaluating pathogenicity of the candidate mutations, confirming the gene function, excluding not associated mutations, choosing the clinically meaningful variants for Sanger Sequencing according to the similarity of clinical presentation is the traditional way to annotate (Jin et al., 2018). However, it is unavoidable that the function and related diseases of the redundant phenotype-unrelated mutants will be analyzed. Here, the phenotype-driven designing “virtual panel” method could automatically filter the genes that is unrelated to the patient’s symptoms, so that the analyser could only focus on the mutations in phenotype-related genes. This method can decrease the genes that should be analyzed, shorten the analysing time and make a more efficient annotation.

Moreover, designing traditional gene panel is a manual work, there might be bias occurring when selecting the gene list in the panel. Also, gene list in produced panel is constant, updating panel aligning with new discoveries is expensive and time-consuming. The virtual panel we run is designed by computer software “Mingjian,” which could avoid the bias due to personal cognition and judgement. In addition, “Mingjian” is according to the database of HPO, OMIM, and HGMD which includes all the known possible genes related to the phenotypes. Since it is actually “virtual,” updating the gene list is not an obstacle. Thus, it could contain all the present discovered, phenotype-related genes. Besides, all the undiagnosed cases can be re-analyzed when more disease-causing mutations are discovered and more linkages between disease and variations are established. Also, every patient has distinct phenotypes, a designed panel may not be applicable for every patient. Phenotype-driven “virtual panel” is based on the phenotypes of the patients, it may simply achieve low-cost individualized analysis when typical and standardized core phenotypes are extracted.

Consequently, we carried out this method in the diagnosis of more patients with neurological diseases to access the diagnostic rate. In 29 cases of patients, 21 of 29 patients were found carrying mutations in related genes. However, according to the inheritance pattern of genes, 2 heterozygous mutations of autosomal recessive genes were excluded. Other 19 of 29 patients were all confirmed with corresponding mutations by Sanger Sequencing.

For the rest of 10 patients who didn’t confirm with the relevant mutations, it may fit one of the following conditions. First, the disease-causing mutations may locate in the undefined genes or genes that have not been experimentally proved to be associated with such neurological diseases. For example, we have found that NCAM1 polymorphisms is associated with autism in a previously undiagnosed case in year 2014 (Zhang et al., 2014). This kind of cases may be solved in the future due to development of research. Secondly, some mitochondrial gene mutations may also be involved but are outside the detection range of Whole Exome Sequencing. The symptoms of most mitochondrial diseases include seizures, mental retardation, developmental delay, metabolic disorders, muscle problems and visual disorders as well (Fang et al., 2017). Both mitochondrial DNA and nuclear DNA mutations may contribute to dysfunction in mitochondria (Liu et al., 2014, 2015; Fang et al., 2017). Therefore, the disease-causing variants in these undiagnosed cases may be located in mitochondrial DNA. Moreover, insertion or deletion which is larger than 50 kb or chromosomal inversion may also cause disease. However, these mutations could not be identified by NGS due to technical limitations. This may not be a rare event since we previously diagnosed a novel DDC gene deletion in the patients who was suspected to carry mutations in DDC gene but only diagnosed with single missense variant (Dai et al., 2018).

Overall, the diagnostic rate in this study was 19/29 = 65.52%, which far exceeds the known diagnostic rate of Whole–Exome Sequencing (25–30%). Therefore, the phenotype-driven virtual panel is an effective method to analyze WES data of neurological disease.

## Data Availability Statement

All the clinical and genetic data of the cases reported in this study have been submitted to the rare disease database, eRAM, at http://www.unimd.org/eram/.

## Ethics Statement

This study was carried out is approved by Capital Medical University Beijing Children’s Hospital Ethics Committee (Ethics Number: 2018-k-63). The protocol was approved by the Capital Medical University Beijing Children’s Hospital Ethics Committee. All subjects gave written informed consent in accordance with the Declaration of Helsinki.

## Consent for Publication

The patient’s parents gave written informed consent to studies and publication of clinical information, images and sequencing data.

## Author Contributions

XW and FF designed the study. XW, FF, and C-HD collected the clinical data. XS, HZ, and Z-HC performed the WES. XS and D-YA analyzed the genetic data. XW, XS, and HZ wrote the manuscript. All authors listed have made a substantial, direct and intellectual contribution to the work and approved it for publication.

## Conflict of Interest Statement

XS, HZ, Z-HC, and D-YA were employed by company Running Gene Inc. The remaining authors declare that the research was conducted in the absence of any commercial or financial relationships that could be construed as a potential conflict of interest.

## Footnotes

1 http://bio-bwa.sourceforge.net/

2 www.broadinstitute.org/gatk

3 annovar.openbioinformatics.org/en/latest/

4 http://omim.org/
